# Preceptorship and Mentorship

**DOI:** 10.1155/2012/790182

**Published:** 2012-07-12

**Authors:** Olive Yonge, Florence Myrick, Linda Ferguson, Florence Luhanga

**Affiliations:** ^1^Faculty of Nursing, Edmonton Clinic Health Academy, University of Albert, 11405 87 Avenue, Edmonton, AB, Canada T6G 1C9; ^2^College of Nursing, University of Saskatchewan, 107 Wiggins Road, Saskatchewan, SK, Canada S7N 5E5; ^3^ Faculty of Nursing, University of Regina, 346 Education Building, 3737 Wascana Parkway, Regina, SK, Canada S4S 0A2

Thirty years ago, there would not have been a special issue on preceptorship and mentorship. Given the rise and consequent research interest in these two practices in nursing, numerous scholarly papers are being published. This particular special edition will be highly relevant to those who participate in preceptorship or mentorship programs from those who coordinate them to the students or graduates who benefit from them.

A number of papers in this publication are the result of research projects. Informal mentoring or teaching will always exist, but when these practices are worthy of research and the research outcomes are applied, replicated in other studies, or simply critiqued, another level of critical thinking emerges. Eventually there will be a solid body of knowledge or evidence that will lead to international standards in the areas of preceptorship and mentorship. F. Myrick et al. reported on the use of evidence-based workshops to prepare preceptors for their role when teaching fourth-year baccalaureate students. Through qualitative analysis, it was established that the workshops were engaging, enriching, and promoted reflection which ultimately benefited the students who were preceptored. C. A. Blum et al. also focused their work on supporting preceptors but did so by developing web-based pod casts which focused on unsafe practice preceptorship issues. The following themes were identified to overcome unsafe practice: welcoming presence, demonstrating empathy, encouraging growth, patience and time as compassionate care, building relationships, and communicating therapeutically. Using focus groups composed of representatives from nursing practice and education, scripts were developed by the researchers and critiqued by preceptors for authenticity. Serendipitously the two groups coming from different perspectives were able to appreciate each others' contributions around the complexities of unsafe practice. S. Boblin et al. also focused on preceptor preparation but for a select population of students international ones. They found four overarching themes that needed to be addressed: culture, knowledge, expectations, and relationships. They then grouped these themes into a model of cross-cultural relationships. An outcome of their research was a manual written to assist preceptors. They noted that this manual could be used for any situation where the student's dominate language is not the one of the current culture or preceptor.

Focusing on the preceptored student experience, K. Ownby et al. compared the traditional clinical experience to a preceptorship experience during their second semester. They randomly assigned seventy one second-year students to a traditional or preceptored group for a 12-week clinical rotation. They found no difference between the two groups on measures of marks, timeliness of clinical paper work, or health education services (surgical medical knowledge) scores.

Researching both preceptors and students, V. Foley et al. examined the intergenerational context. Preceptors are typically older than the students they precept and come from a generation that holds different values. Through the use of phenomenology, she discovered a number of themes and, in this paper, explored “being affirmed.” Essentially this means that there needs to be a culture of openness and respect for generational differences. Generations can learn from each other. On a more cautious note, M. Sedgwick and S. Harris identify challenges inherent in preceptorship programs ranging from issues in the clinical setting to the inadequate preparation of preceptors and faculty. 

D. Jackman et al. focused on rurality. D. Jackman closely examined the literature related to rural nursing preceptorship and how preceptors in rural settings prepared nursing students. She described the opportunities and challenges for learning against a background of what it means to practice in a rural setting. She asked directly what is the impact and meaning of the context for learning. In this case, the context is a rural setting within the health care setting and external to the rural community.

Two papers focused on mentoring. S. Lennox et al. described a program whereby four experienced midwives mentored four new graduates during their first year of practice. They were surprised to find that the new graduates were as much concerned about relationships with each other and staff as they were with acquiring technical knowledge. They were pleased with the effectiveness of using group mentoring. Using groups to mentor versus a one-to-one relationship is worthy of further investigation. The second paper was authored by a cohort of the Faculty Leadership and Mentoring program sponsored by the National League for Nursing and Johnson & Johnson. They provided an overview of a model for excellence in establishing a formal mentoring program for academic nurse educators. If your faculty does not have a mentorship program, this paper is a must read. It is comprehensive and highlights the key areas to establish a successful mentorship program. 

 Overall, you will find this special edition invaluable for the insights and research outcomes the authors have generated. Preceptorship and mentorship programs are not static and evolve with all those who plan and participate in them, and as one author has identified, the context for their delivery is just as critical.

*Olive Yonge* *Olive Yonge* *Florence Myrick* *Florence Myrick* *Linda Ferguson* *Linda Ferguson* *Florence Luhanga* *Florence Luhanga*

